# Towards functional restoration for persons with limb amputation: A dual-stage implementation of regenerative agonist-antagonist myoneural interfaces

**DOI:** 10.1038/s41598-018-38096-z

**Published:** 2019-02-13

**Authors:** Shriya S. Srinivasan, Maurizio Diaz, Matthew Carty, Hugh M. Herr

**Affiliations:** 1 Harvard-MIT Division of Health Sciences and Technology, Massachusetts Institute of Technology, Cambridge, MA 02139 USA; 2 Center for Extreme Bionics, MIT Media Lab, Massachusetts Institute of Technology, Cambridge, MA 02139 USA; 3 Department of Plastic and Reconstructive Surgery, Brigham and Women’s Hospital, Boston, MA 02115 USA

## Abstract

While amputation has traditionally been viewed as a failure of therapy, recent developments in amputation surgery and neural interfacing demonstrate improved functionality and bidirectional communication with prosthetic devices. The agonist antagonist myoneural interface (AMI) is one such bi-directional neural communication model comprised of two muscles, an agonist and an antagonist, surgically connected in series within the amputated residuum such that contraction of one muscle stretches the other. By preserving agonist-antagonist muscle dynamics, the AMI allows proprioceptive signals from mechanoreceptors within both muscles to be communicated to the central nervous system. Preliminary human evidence suggests that AMIs have the capacity to provide high fidelity control of a prosthetic device, force feedback, and natural proprioception. However, AMIs have been implemented only in planned amputations and require healthy distal tissues, whereas the majority of amputations occur in patients who do not have healthy distal tissues. Through the use of a dual-stage surgical procedure which leverages existent tissues, this study proposes a revision model for implementation of the AMI in patients who are undergoing traumatic amputation or have already undergone a standard amputation. This paper validates the resulting AMI’s physiology, revealing robust viability and mechanical and electrophysiological function. We demonstrate the presence of H-waves in regenerative grafts, indicating the incorporation of the AMI into physiological reflexive loops.

## Introduction

Limb amputation is generally perceived as a failure of therapy, instead of an opportunity to provide functional restoration. The traditional approach to extremity amputation suffers from a lack of sophisticated options for patients and results in residual limbs that are frequently complicated by secondary pathologies. Common negative sequelae include bone spurs (14–56%), soft tissue pathology (15–24%), neuroma formation (18–38%), and ulceration (6–31%)^1–3^. These issues, in addition to poor stump formation, often become prohibitive to wearing prosthetic sockets and controlling prostheses and thereby limit motor function. Consequently, there is an alarmingly high rate of revision for amputation, reported between 5–30% for lower extremity (below-knee or above-knee) amputation^4^.

With current biomechatronics and robotic technologies, combined with progressing surgical techniques, we are stepping into an era in which the residual limb can be crafted with dynamic, sensory and motor components to facilitate a smooth transition to a functionally advanced state. Cross-disciplinary work between the biomechatronics and surgery fields has led to the creation of new amputation surgical procedures incorporating neural interfaces through which myoelectric prostheses can be controlled. For example, targeted muscle reinnervation (TMR), regenerative peripheral nerve interfaces (RPNIs), and agonist-antagonist myoneural interfaces (AMIs) address the challenge of deriving stable, high signal-to-noise ratio signals from muscles^5–7^ and enable improved myoelectric control.

AMIs specifically restore dynamically functional neuromuscular constructs to residual limbs and enable natural musculotendinous proprioception by linking agonist-antagonist paired muscles within the amputated residuum^7^. In this configuration, when the user volitionally contracts an agonist muscle, the antagonist muscle undergoes stretch. Length and force information from the antagonist muscle, which are critical for joint stability, fine motor control, and trajectory planning, are conveyed to the central nervous system through afferent signaling pathways^8^. When paired with a myoelectric prosthetic device, force, state, and impedance information from the AMI can be conveyed to control prosthetic joints with high fidelity^9^. Functional electrical stimulation (FES) can be employed to provide feedback to the AMI regarding the prosthesis’ position, state, and impedance^9^. Each muscle of the AMI can be independently stimulated based on parameters calculated from a biophysical model using state, force and impedance data acquired from the AMI. This enables communication of prosthetic movement, whether the joint is moving or locked in position under varying torques. Through these means, the AMI establishes bidirectional signaling between an amputated residuum and a prosthetic device. In murine studies, regenerative AMIs have demonstrated robust efferent and afferent signaling and strains that graded with stimulation amplitude^7,10^. Further, a number of patients have undergone the surgical creation of an AMI during planned amputations. In functional testing, a patient with AMIs outperformed control patients with standard amputation during tasks requiring proprioception^9^.

Multiple AMIs can be created within a residual limb, one for each degree of freedom (DOF) desired in the corresponding prosthetic device. For planned amputations where sufficient distal musculature is healthy and available, AMIs can be constructed by linking the agonist-antagonist muscle pairs associated with the amputated joint through a synovial canal inside the residual limb. However, in the majority of patients requiring amputation, distal tissues are not healthy and/or available for use (in cases of pathology or trauma ~90%). In these cases, the AMI can be constructed using regenerative muscle grafts harvested from a donor site on the same patient. These grafts can be neurotized with transected extensor-flexor pair nerves and linked in agonist-antagonist pairs. Regenerative AMIs demonstrated a time course of maturation compatible with atrophy, neural plasticity, and scarring^7^.

Because the regenerative AMI approach does not require large, healthy, native muscles, it is a versatile approach. Once implemented during a revision operation in patients who have already undergone amputation, it may provide newfound capabilities for myoelectric prosthesis control. The small footprint of each graft (4 cm × 1 cm) enables the method to scale to multiple DOFs. Coupled with the benefit of potentially facilitating advanced motor capabilities through high fidelity control of sophisticated prostheses, implementation of regenerative AMIs in revision amputees may also provide the benefit of neuroma ablation and the potential to sustain muscle bulk due to hypertrophy from volitional use of those muscles. For the aforementioned advantages, regenerative AMIs would be a substantial improvement towards the functional restoration of amputated residua. However, their surgical construction requires the knowledge of the identity/function of each anatomically relevant nerve.

Unlike a *de novo* amputation, a revision surgery procedure is constrained by the tissues in the stump and their structure, as created during the initial amputation. Given the variety of amputation procedures commonly practiced, nerves are often transected at non-standardized locations and may be found buried inside or between muscles or lodged inside bone. Furthermore, neuromas are formed in 13–36% of patients, can inflict pain starting weeks after nerve transection, and are one of the predominant reasons for revision surgery. Whilst most neuromas form as bulbous focal lesions, a majority of them also contain irregular fibers that regenerate in various directions and complicate their isolation. After encapsulation by perivascular tissue, neuromas sprout axons that intertwine and adhere to surrounding tissues^11^. Repeated weight bearing leads to inflammation and irritation, resulting in drastic hyperproliferation (10+ times the original dimensions) and disorganized morphologies^11,12^.

In procedures like TMR and RPNI, the neuroma is removed surgically^13^ and the transected nerve branches are placed in nearby or grafted muscles. Identification of the function and/or target muscle of each nerve is not required, since each nerve innervates one isolated end-target muscle that remains independent of other end-target activation. However, in the case of the AMI, it is necessary to identify the flexor and extensor pairs in order to connect the appropriate grafts and establish proxy joints. Practically, the complex and variable topographical organization of large nerves, compounded by nerve healing and scarring, often makes it challenging to identify specific fascicles, especially at higher levels of amputation.

For instance, the sciatic nerve, is composed of ten major branches, comprising five different flexor-extensor pairs. Efferent activation of the peroneal nerves contracts the tibialis muscle, causing *dorsiflexion* of the ankle joint, while the gastrocnemius undergoes *extension* and transmits afferent signals through the tibial nerve. Though, in principle, the peroneal and tibial nerves could be identified by size and anatomy, after amputation and potential neuroma formation it is often difficult to identify and isolate them with certainty. Lack of definitive nerve flexor-pair identification precludes the construction of regenerative AMIs reflecting each joint.

## Dual-Stage Surgical Paradigm

In this study, we design a dual-stage surgical approach to overcome the challenges related to proper identification of transected nerves and construct AMIs for any revision patient. A detailed flowchart outlining an example case is provided in the supplemental information (Supplemental Fig. 1). Briefly, in the first stage, each terminal nerve in the residual limb would be dissected to the fascicular level and sutured into separate muscle grafts. These grafts would be placed in close proximity to the predicted antagonist graft. After reinnervation, the patient would be instructed to volitionally contract each muscle graft and identify its original motor function. We can also perform ultrasound-guided electrical stimulation of the grafts and ask patients to report the sensed muscle. Each muscle graft would then be tagged using anatomical landmarks or injected fluoroscopic markers. During a second surgery, the appropriate flexor-extensor pairs would be connected to form AMIs. Any grafts that cannot be identified or do not have a clear extensor or flexor function would be left unlinked.

This approach however, involves, interrupting the processes of reinnervation and revascularization and would generate additional scarbeds surrounding the free muscle grafts. This disruption potentially jeopardizes the viability and dynamic functionality (ability to contract/extend) of the AMI, which is essential to the generation of musculotendinous mechanotransduction. Therefore, in this study, we evaluate the ability of the AMI to be formed and function using a dual-stage surgical process, despite additional scarring and a disrupted vascular bed.

We hypothesize that a dual-stage surgery will not mitigate critical functional components of the AMI including reinnervation, tissue health, atrophy and strain when compared to a single-stage AMI (positive control). We hypothesize that forces produced by the AMI will grade with stimulation amplitude. Additionally, we anticipate that regenerative AMI grafts will generate afferent signals and electrophysiological reflex arcs consistent with stimulation amplitude and mitigate atrophy when compared to unlinked grafts (negative control). To evaluate these hypotheses, we construct AMIs in murine models using single (n = 5) and dual stage (n = 5) methods. We also create a negative control group (n = 5) in which regenerative grafts are not linked to form AMIs to investigate the effect of agonist-antagonist pairing on the prevention of atrophy. Five weeks post-operatively, we perform electrophysiological, mechanical and histological studies to evaluate our hypotheses.

## Methods

All animal experiments were conducted under the supervision and approval of the Committee on Animal Care at the Massachusetts Institute of Technology (MIT) in accordance with the relevant guidelines and regulations on male Lewis rats (*n* = 13, weight range: 302–309 g) under 1–2.5% isoflurane used for anesthesia. The experimental (n = 5) and positive control groups (n = 5) underwent AMI construction in a dual-stage and single-stage method, respectively. Additionally, in a negative control group (n = 5), unlinked neuromuscular grafts were created to assess effects of atrophy and afferent signaling in the absence of an antagonist pair.

### Surgical procedure

**Figure 1:**
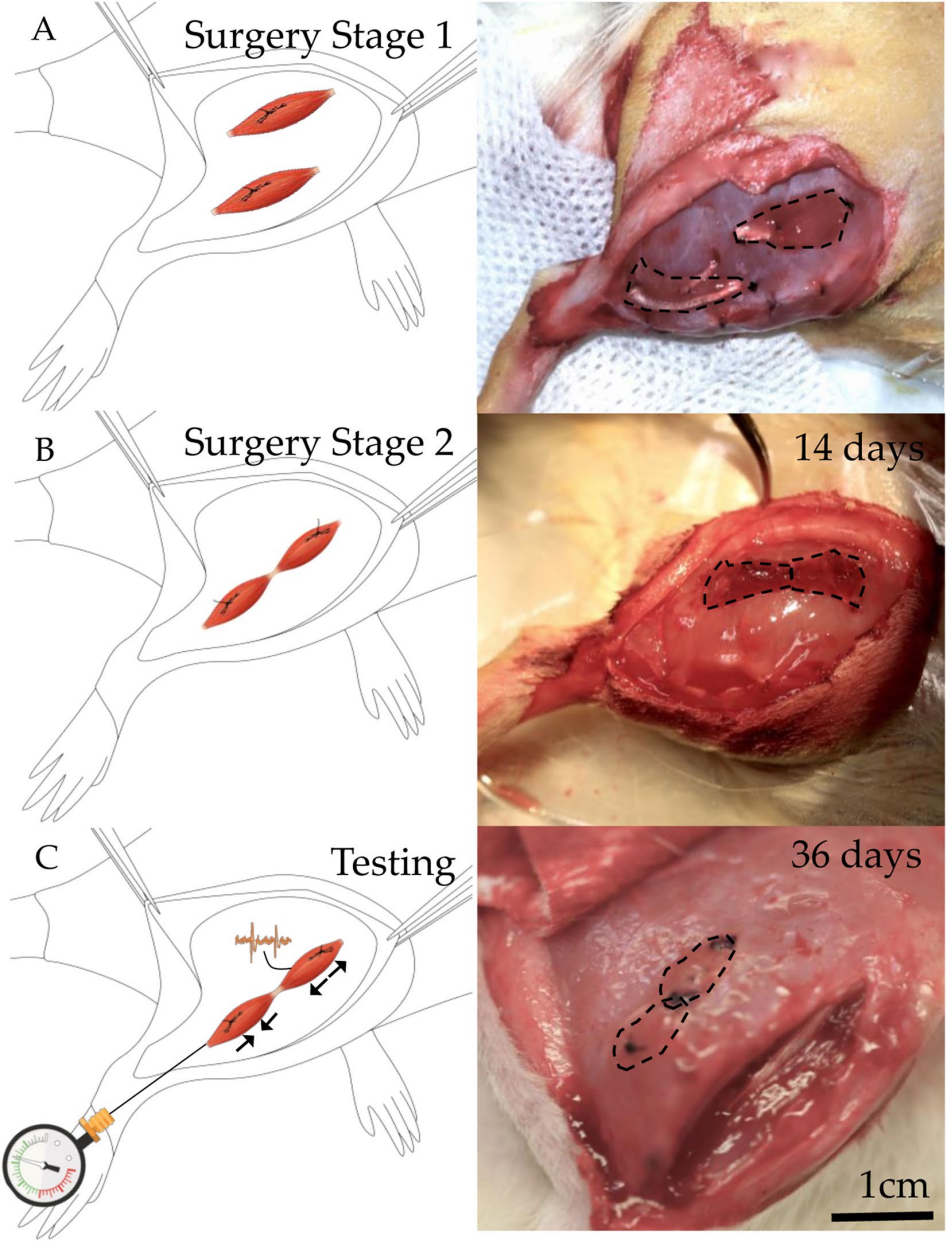
Dual-Stage Regenerative AMI Procedure. (**A**) In the first stage of surgery, two separate regenerative muscle grafts (circumscribed by dotted lines) innervated with the tibial and peroneal nerves are constructed and sutured to the biceps femoris. (**B**) During the second surgical stage, grafts are mobilized from the scar bed, coapted by their tendons to form an AMI, and sutured to fascia. (**C**) After 5 weeks, electrophysiological, histological, and mechanical testing is performed. Under stimulation, the agonist graft contracts while the antagonist undergoes extension. Figure based on original artwork by Stephanie Ku.

In stage one, a curvilinear incision was performed on the right hind limb to expose the anterior and posterior muscle groups. Following partial reflection of the biceps femoris, the extensor digitorum longus muscle was isolated and disinserted. The weight and length of the muscle were measured. Myotomy of the extensor digitorum longus muscle created two equally sized muscle grafts. Muscle grafts were sutured to the superficial fascia of the biceps femoris (5–0 absorbable suture) and situated approximately one centimeter from each other. The tibial and common peroneal nerves were isolated, distally transected, and elevated through slits in the biceps femoris. Their usage simulates that of any given flexor-extensor pair or two unknown fascicles in the human case. A superficial myotomy was performed through the epimysium of each muscle segment to neurotize with either the peroneal or tibial nerve (8-0 microsuture) and create two distinct innervated muscle grafts (Fig. 1A). The incision was closed in layers using a 4-0 suture. Two weeks after the first surgery, we performed electromyography to confirm partial reinnervation. In the murine model, the nerve innervating each graft was known. However, in the human implementation, electromyography would be performed to assess the function of each graft and thereby derive the identify of the innervating nerve. Then, during a second surgery, following an incision of the area overlaying the grafts, blunt dissection of the scar bed surrounding each muscle was performed to mobilize the grafts. The tendinous regions of both grafts were linked using 4–0 nylon sutures to create an AMI. The ends of the AMI were then sutured to the fascia of the biceps femoris. The incision was closed using a 4-0 suture.

### Controls

For positive controls, AMIs were created out of regenerated muscle grafts in a single surgical step, as previously reported^7^. In negative controls, the second stage of surgery was not performed and muscle grafts remained isolated throughout (Supplemental Fig. 2).

### Outcome Assessment

To assess reinnervation, insertional needle EMG was performed weekly as described previously^7^. Six weeks post-operatively, electrophysiology was performed to measure ENG and EMG from each nerve and muscle, respectively. Excursion, insertional activity, atrophy, coupled motion and strain were quantified as previously described^7^. Procedural details can be found in the supplemental information.

In dual-stage AMIs, the forces generated by the agonist graft were measured using a Shimpo FGV1XY 1lb capacity digital force gauge. 4–0 nylon suture was sutured to the end of the agonist muscle and attached to the lever arm of the force gauge. The system was positioned such that a small non-zero force was registered on the system at baseline to ensure that no slack was present during the measurement. Stimulation was applied to the agonist graft at increasing amplitudes and output forces were recorded. 

Following euthanasia, the AMI and innervating nerves were harvested and fixed in 4% paraformaldehyde for 24 hours, paraffin processed, embedded, and sectioned at 5 μm. Hematoxylin & eosin (H&E) and trichrome staining were performed on at least five slices per animal to assess morphology, healing, revascularization, reinnervation, and fibrosis. Additionally, alpha bungarotoxin (ab120542, Abcam) was used to stain for synaptogenesis and verify the extent of reinnervation. S46 (Developmental Studies Hybridoma Bank) binds to slow, tonic, myosin heavy chains and was used to stain intrafusal spindle fibers (1:200 dilution in blocking buffer). A goat anti-mouse green fluorescent protein secondary antibody was used to visualize the s46 binding. Immunofluorescence images were taken on an Evos FL Auto epifluorescencemicroscope (Fisher) with identical lighting conditions.

For all analyses, t-tests were used to compare the experimental and control groups to evaluate statistical significance. Prior work and comparable studies demonstrate normality in the measured metrics.

## Results

In all animals, AMIs were effectively created employing the peroneal and tibial nerve pair using a two-step surgical process. At terminal harvest, gross morphology indicated well-healed and revascularized grafts. No infection, graft failure, necrosis or ulceration occurred in any animals.

### Atrophy

**Figure 2:**
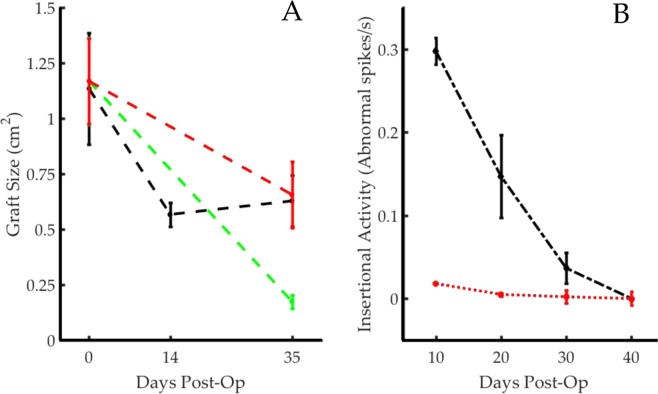
Reinnervation and Atrophy Trends. (**A**) Average graft size (n = 5 each) and standard deviation at each surgical step or final testing session is shown; dual-stage AMIs (black) underwent similar rates of atrophy to single-stage AMIs (red) but were significantly less atrophied when compared to the separated grafts (green). (**B**) Insertional needle EMG activity levels in the dual-stage AMI (black) are compared to those in the contralateral gastrocnemius (red) (n = 5) and demonstrate reinnervation of the grafts over the course of 40 days.

It is important to maintain muscle mass in AMIs, since it directly correlates with strength of efferent signal. For dual-stage AMIs, muscle atrophy occurred predominantly between stage one and stage two, during the reinnervation and revascularization process (51 +/− 2.5% reduction in size). The average total atrophy 35 days after the first surgery was 41.62 +/− 3.2%. Notably, between the creation of the AMI and the terminal harvest, animals demonstrated a 9.7 +/− 1.8% increase in graft size (Fig. 2A). Single-stage AMIs underwent 52 +/− 7.1% atrophy over the course of 35 days. The magnitude of atrophy between the single-stage and dual-stage surgery groups was insignificant by a one-tailed 2-sample t test at p = 0.01. However, unlinked grafts experienced an 87 ± 4.2% reduction in size, and were significantly more atrophied compared to linked AMIs at the p = 0.01 level (one-tailed 2-sample t test). Supplemental Figure 2 exhibits muscle grafts upon creation and the atrophied outcome in a representative animal with unlinked grafts. The stark contrast between the unliked control grafts and linked AMI grafts evidences the advantageous function of the agonist-antagonist linkage in preserving and potentially promoting muscle mass.

*Reinnervation* was assessed through needle-based electrophysiology. The frequency of abnormal insertional activity decreased steadily indicating gradual reinnervation of the regenerative muscle grafts. 35 days post-surgery,innervation of the experimental grafts was comparable to that of the contralateral gastrocnemius muscle (p < 0.001, 2-sample t-test) (Fig. 2B). This result is comparable with previously published trends^7,14^. During the reinnervation process, end plate spikes, polyphasic motor unit potentials and fibrillation potentials commonly occurred.

### Efferent and Afferent Signaling

**Figure 3:**
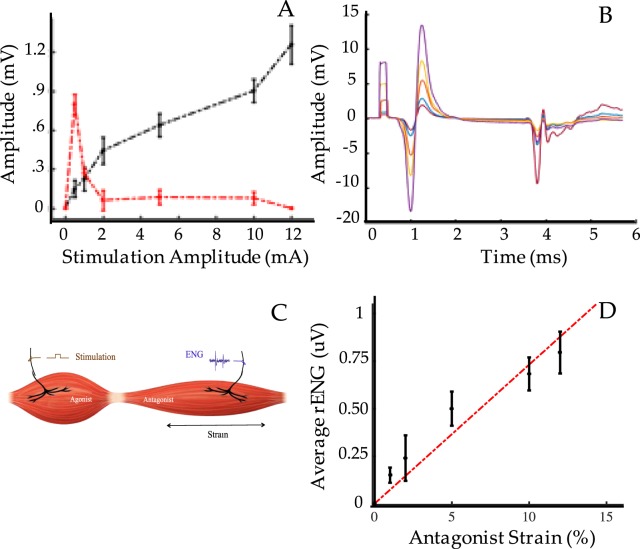
M-wave and H-wave Trends. (**A**) Shown are average m-wave (black) and h-wave (red) amplitudes at each stimulation amplitude (0.5, 1, 2, 5, 10 mA). (**B**) Shown are average EMG amplitudes of m and h waves in response to 100 us, squarewave stimulation pulses on the tibial nerve (n = 150). As m-wave amplitude increases, h-wave amplitude decreases. (**C**) Electrophysiological set up to measure afferent signals generated from the antagonist due to agonist contraction. A stimulation pulse is applied to the agonist nerve while measuring ENG from the antagonist nerve to survey for afferent feedback signals. (**D**) Graded afferent signals correlated linearly with antagonist strain demonstrating that the agonist and antagonist grafts performed proportionally coupled excursion and appropriate mechanotransduction.

 Isolated efferent electromyographic signals with high signal-to-noise ratios are required for the control of a prosthetic device. Using neural stimulation, isolated EMG signals were produced in each muscle graft, with no electrical leakage or inappropriate noise in the antagonist graft. The average signal to noise ratio was 80.2 for dual-stage grafts, 84.0 for single-stage grafts, and 69.3 for unlinked grafts, which is sufficient for myoelectric prosthesis control^5^. Despite the scarring present, signals were isolated and produced a healthy biphasic trend. Thirty-five days post-operatively, muscles produced strong m-wave responses, and compound muscle action potentials that graded with stimulation amplitude (Fig. 3A). In addition, h-waves were identified in 70% of the dual-stage AMI grafts (Fig. 3B) during stimulation of the peroneal or tibial nerves. These responses were elicited at stimulation of 0.5 mA and decreased in magnitude through 5 mA. 10 mA stimulation did not elicit any h-waves in 8 of the 10 cases tested. Notably, h-waves were not detected in unlinked grafts. H_max_/M_max_ values ranged between 5.32 at 0.5 mA stimulation and 0.076 at 10 mA stimulation, comparable to previously established values ^15^. Afferent signals on the antagonist nerve were produced in response to stimulation on the agonist nerve (Fig. 3C) with magnitudes and latencies similar to those previously reported^7^. The afferent signals (rectified EMG) graded with the strain generated in the antagonist muscle (Fig. 3D) – a direct result of proportionally coupled excursion. The main functionality of the AMI is to produce graded efferent and afferent signals in response to increasing agonist contraction. The electrophysiological results above confirmed that the AMIs created through a dual-stage process were capable of graded efferent and afferent with no significant differences from single-stage AMIs.

### Force Production

**Figure 4:**
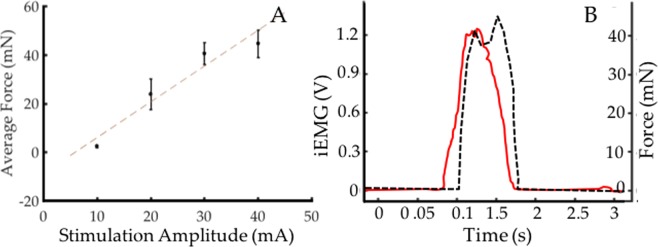
Force Production. (**A**) Forces produced by the muscles linearly scale as a function of stimulation amplitude. (**B**) Representative plot of iEMG (red) and force (dotted black) production shows physiologic excitation-contraction coupling delay.

Force feedback, via activation of Golgi tendon organs, is a critical component of the AMI. The ability for dual-stage AMIs to produce force was measured using a force gauge during electrical stimulation of the innervating nerves. Forces produced in the agonist muscle, ranged between 19.6–58.8 mN, and linearly correlated with stimulation amplitude and integrated EMG. Linear regression yielded an r^2^ = 0.86 (Fig. 4A) for this correlation. Integrated EMG and force production from a representative test is shown in Fig. 4B. In their linear configuration, the regenerative AMIs had an electromechanical delay (between EMG production and force generation) of 59 +/− 7 ms on average. At lower stimulation amplitudes (1 or 2 mA), this delay was greater, ranging from 79–120 ms. This is consistent with ranges of skeletal muscle under concentric or eccentric contraction in humans^16,17^, suggesting that the AMI’s mechanotransduction kinetics are appropriate for its intended purpose of providing proprioception.

### Strain Generation

The primary component of afferent feedback is spindle activity, generated by strains in stretching muscle in response to agonist contraction. These grade with stimulation amplitude in regenerative AMIs^7^. Here, the addition of a separate surgical step did not adversely affect the strains produced by the regenerative AMI through excessive scarring. Strains in the agonist and antagonist are inversely proportional and represent proportionally coupled motion (Supplemental Fig. 3). The average strain was measured to be 10.7 +/− 3.1%. Control AMIs created through a single stage surgery performed with 8.1–1.82% strain, which is not significantly different from the experimental dual-stage group (p = 0.05, 2-sample t-test). No coupled motion occurred in unlinked grafts in the negative control group as predicted.

### Histological Results

**Figure 5:**
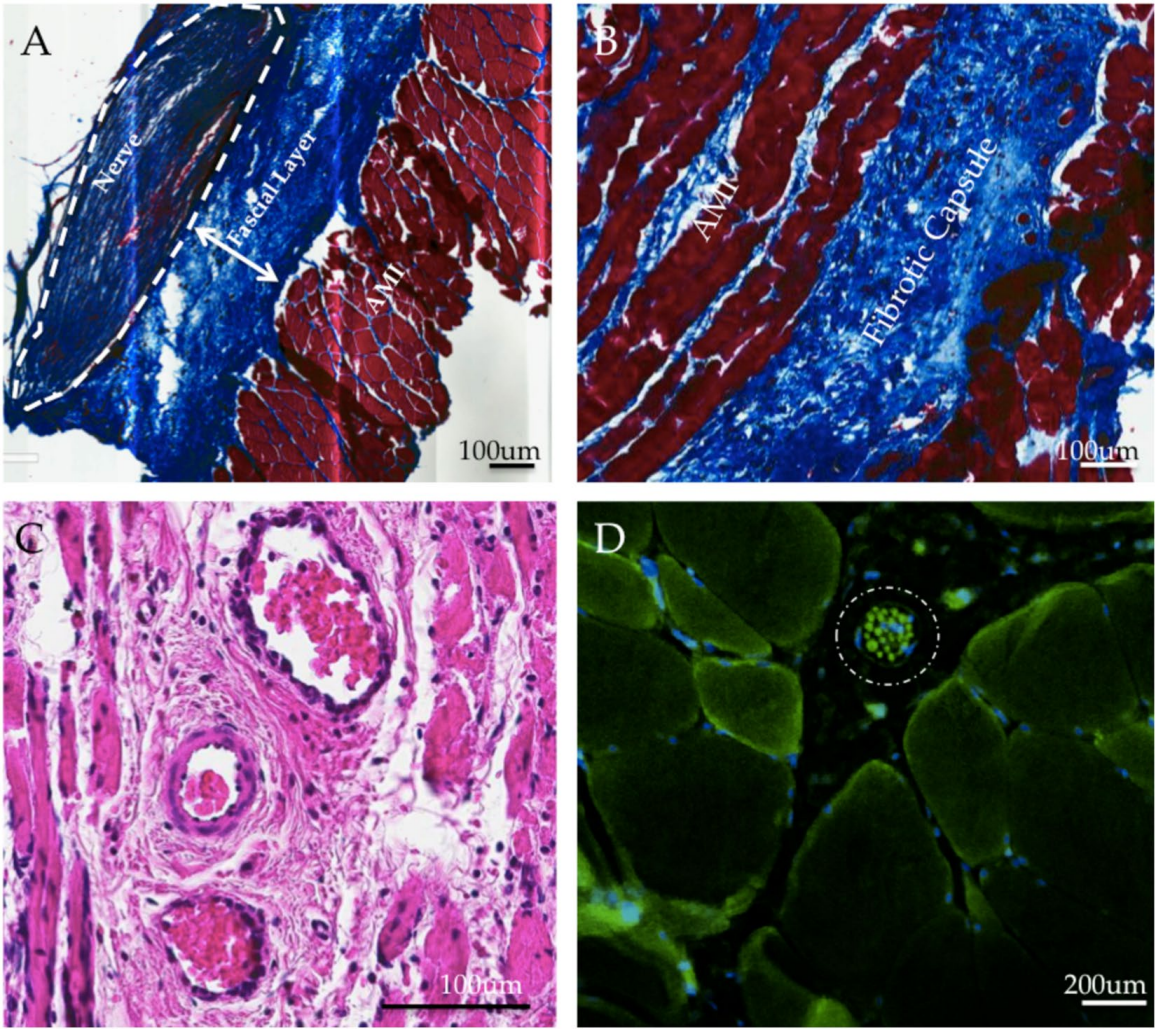
Histological Results. (**A**) Well-demarcated partition between the fascial layer and AMI. This representative slice captures a healthy collagen-bound nerve entering the muscle layer and reinnervating the muscle. (Trichrome stain). (**B**) The fibrotic capsule surrounding the upper layer of the AMI, through which new vessels formed, is comprised of loose, anisotropically oriented collagen. (Trichrome stain). (**C**) Well-epithelialized angiogenic vessels through scar bed facilitate revascularization. Surrounding musculature appears healthy and mature. (H&E stain). (**D**) A spindle fiber (circled) is shown containing numerous intrafusal fibers between motor unit groups (s46 stain).

Dual-stage AMIs were encapsulated in a collagenous fibrotic capsule. The tendon-tendon coaptation showed durable healing, with myocytes aligned isotropically near the region of coaptation and transitioning to collagenous tendon (Supplemental Fig. 4). The fascial layer between the AMI and biceps femoris measured 176 +/− 26 μm in the dual-stage (Fig. 5A) AMI and 169 +/− 38 μm in single-stage controls, an insignificant difference (t = 0.21, p = 0.41). The fibrotic capsule (Fig. 5B) thickness measured 335 +/− 73 μm in dual-stage AMIs and 274 +/− 83 μm in single-stage controls, an insignificant difference (t = 1.25, p = 0.11). Collagen patterns and luxol fast blue staining demonstrated well-myelinated nerve branches spreading throughout the muscle. Muscle tissues were healthy and comprised of both regenerating myocytes and fully mature myocytes (Fig. 5C). The average fiber thickness was 84 +/− 9 μm in AMI grafts and 89 +/− 7 μm in control grafts, an insignificant difference (t = 0.98, p = 0.35). In the second surgery, despite disruption of the scar bed through which angiogenesis occurred, both small capillaries and larger epithelialized vessels were formed throughout the graft, predominantly entering through the underlying scar bed. Blood vessels were found alongside the innervating nerves (Supplemental Fig. 5) and provided a non-disrupted vascular supply during the second transfer.

Alpha bungarotoxin staining revealed normal synaptogenesis, centered near the region at which the nerve was sutured into the muscle. Intrafusal spindle fibers are required for the transduction of critical fascicle length information for proprioceptive signaling. Muscle cross sections showed viable spindles with 6–16 intrafusal fibers each when stained with s46 (Fig. 5D).

## Discussion

There are limited strategies to avail bidirectional efferent-afferent prosthetic control to persons with limb amputation. Of these, the AMI uniquely includes the provision of naturally-generated muscle-based proprioception^9^. This is accomplished through regenerative grafts linked in an agonist-antagonist conformation, which lend  themselves to robust efferent and afferent signaling^7,10^. The isolation of end-target function in the AMI architecture make it particularly well-suited for direct neural control of prostheses. In contrast, standard amputation and TMR require post-facto methods such as pattern recognition to identify the neural composition innervating end targets.

Due to limitations of signal processing, pattern recognition has provided limited function for many patients, resulting in a limited acceptance rate of pattern recognition-based devices^18,19^. Moreover, other amputation techniques such as TMR^18^ and RPNI^14^ employ no agonist-antagonist architecture and therefore do not provide the capacity for natural muscle-tendon proprioception. In contrast, the regenerative AMI can return lost functionality to those with standard amputations by providing bidirectional signaling capabilities for use in consort with myoelectric prostheses^7^.

### Outcomes of the Dual-Stage Approach

The ability to create, resituate, and assemble regenerative peripheral nerve grafts is critical for the implementation and scaling of regenerative AMIs for multiple DOFs in revision amputations. To emulate an amputation model, dissected branches of the sciatic nerve were placed into separate regenerative muscle grafts. Two weeks later (after identifying the function of each graft under stimulation), individual flexor and extensor grafts were paired. Mechanical, electrophysiological and histological evidence showed that a dual-stage surgery was viable in creating AMIs capable of graded afferent/efferent signaling at high SNRs. Strain rates, atrophy, and reinnervation were not negatively impacted by a dual-stage approach. Graded force production further validated the ability of the dual-stage AMI to overcome any limiting contractile conditions of the muscle, including elasticity/compliance constraints due to scarring and minimized fiber length from surgery^19^.

### Important Considerations for Revascularization

The disruption of vascular beds and added scarring through a second surgical stage proposed a significant viability concern in terms of vascularization. The revascularization of regenerative grafts occurs through a three-stage process: (1) plasmatic imbibition, (2) inosculation and capillary ingrowth, and (3) revascularization. Immediately following whole skeletal muscle graft transfer, diffusion-based imbibition comprises the primary mode of nutrient and chemical exchange. 24 hours after grafting, previous studies have reported ischemic degeneration of large vessels within the muscle. Three to ten days post-operatively, new vessels grow through the scar bed into both old vessel lumens as well as newly formed vessels^20^. In this study, 14 days post-operatively, grafts in the dual-stage experimental group were re-harvested, destroying any capillary ingrowth, restarting the revascularization process and risking the viability of the grafts. However, both functional and histological results demonstrated insignificant differences between the control and experimental groups in terms of atrophy or myocyte morphology. Numerous small-diameter vessels were found growing centripetally through the scar bed to revascularize the graft. Moreover, it is likely that the blood vessels adjacent to the innervating nerve, which were not disrupted during the second surgery provided a vascular supply that helped mitigate ischemia during the second transfer. In addition, the angiogenic processes activated by the first transfer, consisting of various elevated mechanical, hormonal and metabolic components^21^, remained active at the time of the second transfer and would have aided in the second revascularization process of the AMI.

### Integrating with Reflexive Loops

In addition to validating a modular surgical approach for revising amputation limbs, this study demonstrated that regenerative muscle architectures are capable of recreating proprioceptive reflex loops. Given the recency of AMIs, which enable proprioceptive signaling through afferent neural pathways^7,10^, there is a need to validate the presence and activity of reflexive loops in this architecture. Clinically, the Hoffman reflex (indicated by h-waves) is used to study motor circuits, muscle tone, conduction pathologies in afferent axons, and diagnose peripheral neuropathies^22,23^. H-wave activity reflects the response of afferent fibers and reflex loops^15,24^. In this study, stimulation of effect nerves yielded h-waves for 7/10 grafts in the dual-stage AMI group. The amplitudes of these waves decreased as stimulation amplitude approached the supramaximal amplitude. This pattern is characteristic of healthy reflex loops. Lack of activity in certain grafts can be attributed to anesthetic states, varied recording electrode placement, and destructive interference of orthodromic and antidromic impulses due to the placement of the stimulation electrode. Nevertheless, the integration of regenerative muscle grafts in natural reflexive loops indicates the ability of the regenerative AMI to be integrated into the nervous system, a newfound benefit of the AMI. This result is not only important for the amputation model, but also opens avenues for the creation of similar regenerative architectures to manage afferent neuropathologies. Future work should focus on further characterizing the reflexive loops, especially in the 0–1 mA stimulation range where they are gradually expected to increase in magnitude. Insights on the reflexive mechanisms enabled by the unique AMI architecture will play a role in the design of neuromuscular models operating on neuroprostheses that are used in consort with the AMI.

### Mechanical Linkage Influencing Atrophy

This study additionally provides valuable insight on the use of agonist-antagonist pairs for the potential prevention of disuse atrophy. In unlinked grafts, the high rate of atrophy (90%) compared to that of linked agonist-antagonist grafts (50%), suggests that mechanical linkage strongly influences the process of atrophy. This may be due to afferent signaling from reciprocal innervation, which promotes volitional use and consequent hypertrophy. This is not only important in the context of this surgery, but also in any surgeries or traumatic injuries where disuse atrophy ensues due to disruption of agonist-antagonist muscle pairs^25^.

### Translation to Amputation Surgery

For patients undergoing revision of a stump, this study presents a modular approach to assembling regenerative AMIs for each desired DOF. These DOFs can be controlled by an advanced bionic device using a controller calibrated to the signals generated from each muscle graft in the AMI^26^. In the worst-case scenario, where the identity of each nerve fascicle is uncertain, fascicular splits could be performed to divide as many fascicles as technically feasible. Each fascicle would then be placed inside a muscle graft and secured onto fascia, close to its estimated muscle pair. After reinnervation, the patient would be instructed to volitionally contract each muscle graft and identify its motor function. Each muscle graft would then be tagged using anatomical landmarks or injected fluoroscopic markers. During a second surgery, the appropriate flexor-extensor pairs would be connected to form AMIs. If insufficient pairs or odd numbers of grafts existed, two flexors could be connected to one extensor or vice versa. For example, distinct branches of the tibial nerve innervate the plantaris and soleus, both of which help to plantarflex the ankle joint. Both flexor tendons could be connected to the extensor, the tibialis anterior, that dorsiflexes the ankle joint (Supplemental Fig. 6). Nerves for which corresponding DOFs are unavailable/undesired (fixators and synergistic muscles) in the prosthetic device could remain as isolated regenerative grafts in the residuum. In the best-case scenario, as in the case of a planned amputation, one or two natural agonist-antagonist pairs could be disinserted from the articulating joint and repositioned through a synovial canal around the bone. Then, regenerative grafts could be created for other smaller nerves that were transected and connected to their known antagonists, or placed on superficial fascia for future action. By tailoring a combination of native and regenerative AMIs, each residuum could enable afferent signaling and improved controllability of a prosthetic device.

### Considerations for Scale

Translation and implementation in humans will require a proportional scaling of various components in this surgical approach, including graft size, surgical time course, and anatomical placement. In rats, we limited the size of the grafts to 150 mg, which has been reported to be the critical limit beyond which the surface area to volume ratio exceeds diffusional capabilities^21^. In humans, maximum free graft size has been reported to be 1 cm × 4 cm for similar applications^27^. Additionally, the time course of reinnervation in humans will occur on the order of months, instead of weeks. Coupled with post-surgical edema, scarring, wound healing, and other considerations, which limit a secondary operation within 60–90 days, we predict the second stage of surgery to take place between 3–6 months after the first surgery in humans. Finally, the availability of superficial fascia and the shape of the residual limb will dictate the distance between grafts during the initial surgery.

### Summary of Benefits

The regenerative AMI’s direct integration with natural nervous-signaling circuits, isolation of target efferent signals with high SNR, scalability, and adaptability to a broad range of pre-existing anatomical configurations make the demonstrated method valuable for stump revision. While the proposed dual-stage approach requires two separate surgical interventions, the outcomes of this study suggest that the associated risk of graft degeneration/failure are fairly low and that the process results in an AMI with robust bidirectional signaling capabilities, potentially providing improved functionality for patients with amputation. There is precedent in the literature and in practice for various multi-step surgeries, including sympathectomies^28^, tumor resections^29^, fistula corrections^30^, and hip replacements^31^. Further, previous studies demonstrate that revision surgery in persons with amputation often promotes favorable outcomes including pain relief and improved motor function^3,32^.

Despite the volume of patients suffering from stump-related problems, there are few standardized protocols for stump revision and functional restoration^33^. This study offers a practical and technically feasible surgical strategy for implementing the regenerative AMI through a dual-stage method to revise amputated residua and return lost nerve function for at least some, if not all, terminal nerves. This work represents a step towards restoration of natural dynamic functionality, allowing persons with amputation to benefit from improved prosthetic control and natural proprioceptive feedback.

## Supplementary information

Supplemental Information

## Data Availability

The datasets generated during and/or analyzed during the current study are available from the corresponding author on reasonable request.
