# Supplementary material for: Towards functional restoration for persons with limb amputation: A dual-stage implementation of regenerative agonist-antagonist myoneural interfaces
Source: Sci Rep. 2019 Feb 13;9:1981. doi: 10.1038/s41598-018-38096-z (PMC6374452; doi:10.1038/s41598-018-38096-z)
Supplement: Supplementary file 1 — Supplemental Information [file 41598_2018_38096_MOESM1_ESM.pdf]

Supporting Information

Towards functional restoration for persons with limb amputation: A dual-stage implementation of regenerative agonist-antagonist myoneural interfaces

Shriya S. Srinivasan, BS.[1,2], Maurizio Diaz[2], Matthew Carty, MD[3], Hugh M. Herr, PhD[2]

[1]Harvard-MIT Division of Health Sciences and Technology,
Massachusetts Institute of Technology, Cambridge, MA 02139, USA

[2]Center for Extreme Bionics, MIT Media Lab,
Massachusetts Institute of Technology,
Cambridge, MA 02139, USA.

[3]Department of Plastic and Reconstructive Surgery,
Brigham and Women's Hospital,
Boston, MA 02115, USA.

Correspondence can be sent to hherr@media.mit.edu

Supplemental Methods:

Numerous electrophysiological, histological, and mechanical assessments were performed to evaluate the function and recovery of AMIs created through a single and dual-stage process. Here, we outline the procedures used to acquire these data.

Insertional EMG to assess reinnervation

To identify the extent of reinnervation in the muscle grafts, insertional EMG was performed. The regenerative muscle grafts were identified by palpation or through a small incision in the hindlimb, thereby exposing the grafts. A monopolar (30-gauge, Natus Medical) needle was placed into the muscle graft and stabilized using an external clamp. This prevented any shifting of the electrode, which could have cause penetration into the biceps femoris. If palpation was used to identify the graft, after insertion of the needle, a small stimulus (100 us, 0.5 mA) was applied on the stimulating needles to ensure placement in the graft and not in the underlying biceps femoris.

Electrophysiology to measure efferent and afferent signals

Electrophysiological testing was performed six weeks after the first operation. We assessed the ability of the AMIs to provide stable, isolated, efferent control signals for prosthetic device modulation. We also evaluated their ability to generate natural proprioceptive afferent signals. Skin was incised and pulled back to expose the AMIs or unlinked muscle grafts. 30-gauge bipolar needles were inserted into each muscle and connected to a differential biopotential amplifier (20kS/s sampling frequency, 16-channel amplifier stage with 200x gain, Intan Technologies).

In the first set of tests, the innervating nerve of each muscle was independently stimulated using a hook electrode with pulses generated from an NL800 Current Stimulator (Digitimer) ranging between 0.5 and 12 mA. Pulse width was held constant at 100 us. Efferent signals from both muscles were recorded to map the stimulus-intensity and efferent EMG response. Given the tendon-tendon coaptation, for a given nerve, we expected efferent signals resulting from the agonist, but electrical silence from the antagonist muscle.

In a second set of tests, we performed afferent ENG recordings. The agonist nerve was stimulated using a hook electrode and ENG from the antagonist nerve was recorded. Details of afferent signal recording and processing can be found in Srinivasan et al. (Science Robotics, 2017). Stimulation on the agonist nerve caused contraction of the agonist muscle stretching the antagonist. Consequently, afferent feedback signals were generated in the antagonist and recorded on the antagonist nerve. We analyzed data to reveal gradation in afferent activity proportional to stimulation intensity. All EMG and ENG signals were processed in MATLAB.

Stain generation and excursion

During the aforementioned tests, video recording of the excursing muscle grafts was performed using a Nikon D3200 DSLR camera. This captured the 2D motion of the constructs from an angle that was orthogonal to the plane of the underlying biceps femoris. Frames capturing the resting and maximal contraction points of the antagonist grafts were used to quantify the percent strains and excursion values induced on the agonist at each stimulation amplitude. These values were used to assess whether the AMI's linkage of agonist-antagonist muscles created proportional responses in the antagonist muscle to agonist contraction. Further, strain values were compared to those of physiological, biologically intact muscles to ensure that resulting afferent signals would mimic those naturally received by the central nervous system.

Atrophy

During implantation and the terminal procedure, the muscle grafts were photographed using a Nikon D3200 DSLR camera. The border demarcating the ends of the graft was outlined using surgical marker and used for quantification during data analysis. Furthermore, grafts were weighed upon explant. These data were synthesized to determine the effect of a dual-stage surgical process on the atrophy of the muscle grafts comprising the AMI.

Supplemental Figures:

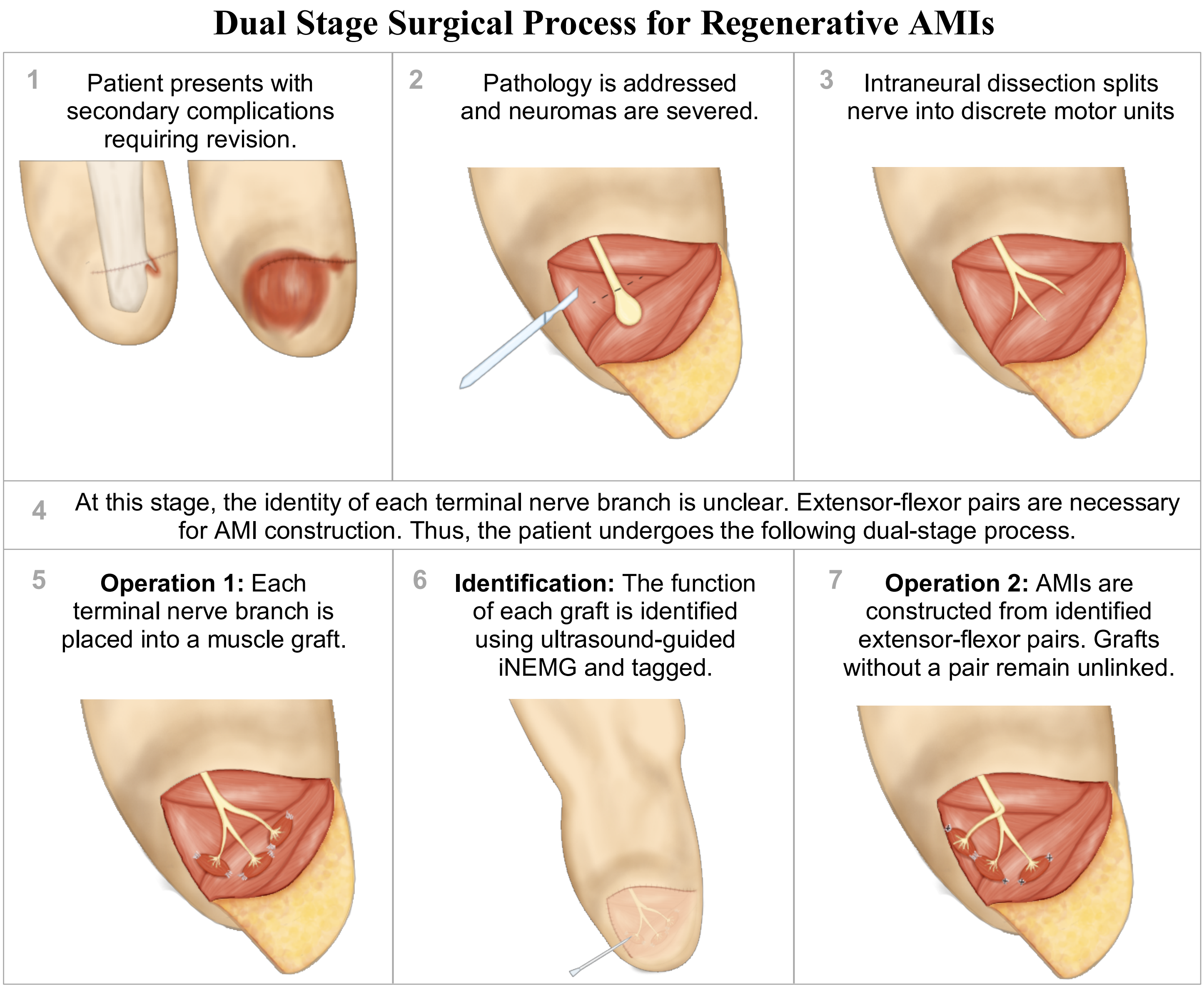

Supplemental Figure 1. Dual-stage Surgical Process to Create AMIs during Revision Surgery. Flowchart describes a sample case in which (1) a patient would present for secondary complications of amputation requiring revision surgery. Consistent with the current surgical process, (2) neuromas would be severed and buried in between muscle without a target end organ. These nerves can easily form into painful neuromas. Furthermore, myoelectric control from these nerve endings is challenging because the signals are difficult to source and are often contaminated by surrounding muscles. During revision, the dual-stage process we propose would be employed to create regenerative AMIs using transected peripheral nerves. (3) In the revision surgery, an intraneural dissection would yield numerous terminal motor units. (4) However, the function of each of these nerves would be potentially unknown. The creation of AMIs requires agonist-antagonist pairs. The following dual-stage surgical process enables their identification. (5) During the first revision operation, each terminal motor unit would be placed into a regenerative muscle graft. After a brief period of reinnervation, (6) the function of each graft can be ascertained through patient reporting during ultrasound-guided muscle stimulation or volitional contraction. The grafts can be labeled with radiopaque markers or identified using anatomical landmarks. (7) During a second operation, grafts that are found to be extensor-flexor pairs will be linked to form an AMI. Grafts

that do not have a pair, cannot be identified, or do not have solely a flexor or extensor function will be left unlinked. Figure adapted from original artwork by Stephanie Ku.

As compared to the current amputation paradigm in which the function of all these transected nerves are lost, the dual stage process provides the opportunity for the functionality of at least some transected nerves to be preserved and utilized for myoelectric control and naturally-generated proprioception.

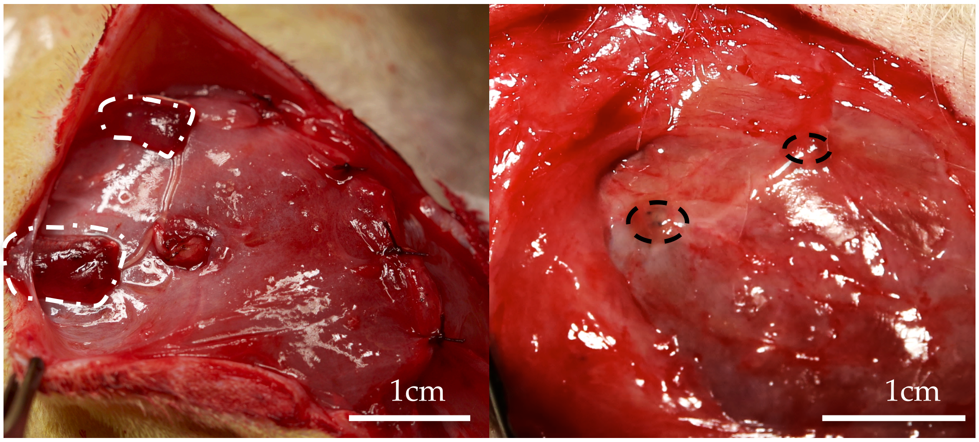

Supplemental Figure 2. Progression of Graft Volume. (Left) Photograph capturing surgical construction of regenerative muscle grafts in the first operation. Grafts, outlined in the dotted lines, were placed on the biceps femoris and innervated with the peroneal and tibial nerves. (Right) In the negative control group, regenerative nerve grafts remained unlinked throughout the course of the experiment (36 days), resulting in ~90% atrophy, no afferent signaling, no reflexive signals and immeasurable strains. The comparison between linked and unlinked regenerative grafts evidences the beneficial nature of the agonist-antagonist linkage in preserving and potentially promoting muscle mass.

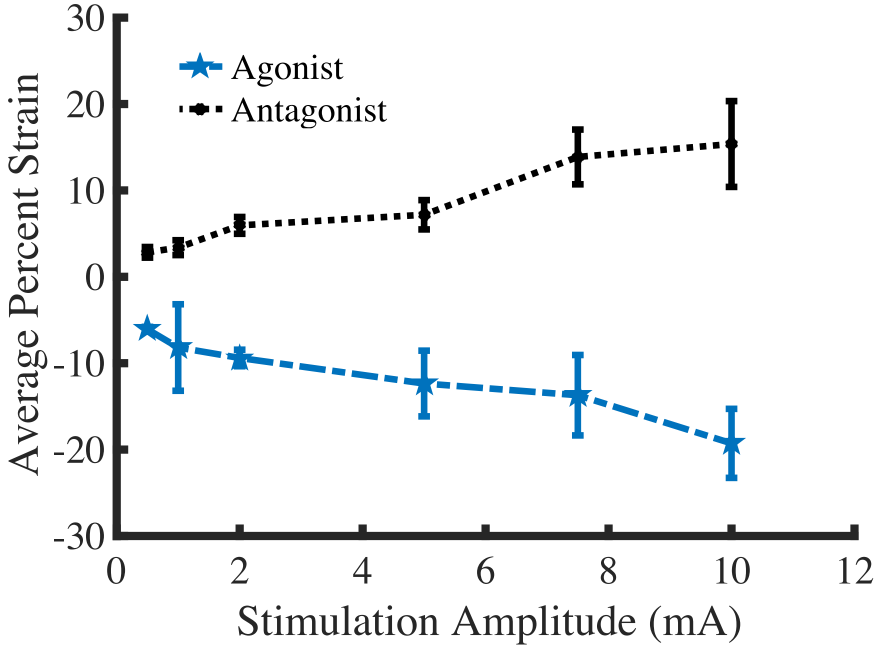

Supplemental Figure 3. Couple Strain Generation. Strains generated in each muscle during stimulation of the agonist are plotted. Strains generated by the agonist and antagonist muscles are complementary. Each point represents the average and standard deviation of n = 5. As demonstrated by the trend line, the strains grade proportionally with stimulation amplitude and validate the coupled nature of excursion in the mechanically linked AMI grafts.

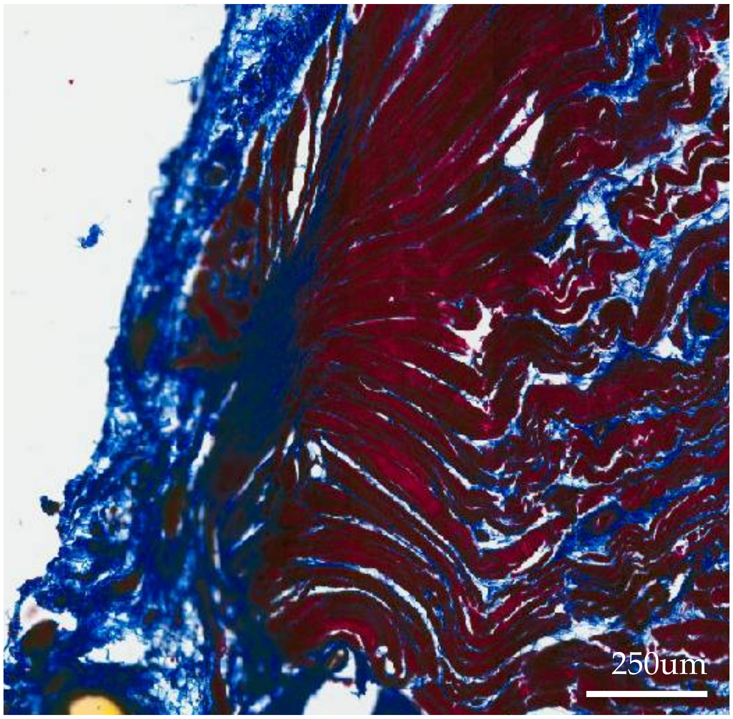

Supplemental Figure 4. Morphology of Tendinous Coaptation. A cross section of muscle near the tendon-tendon junction is trichrome stained. Myocytes (red) are isotropically aligned and merge healthily with collagenous tendon at the site of coaptation. This suggests that the healing process formed a strong bridge that would enable the translation of tension across the tendon-tendon bridge between the two muscle grafts comprising the AMI.

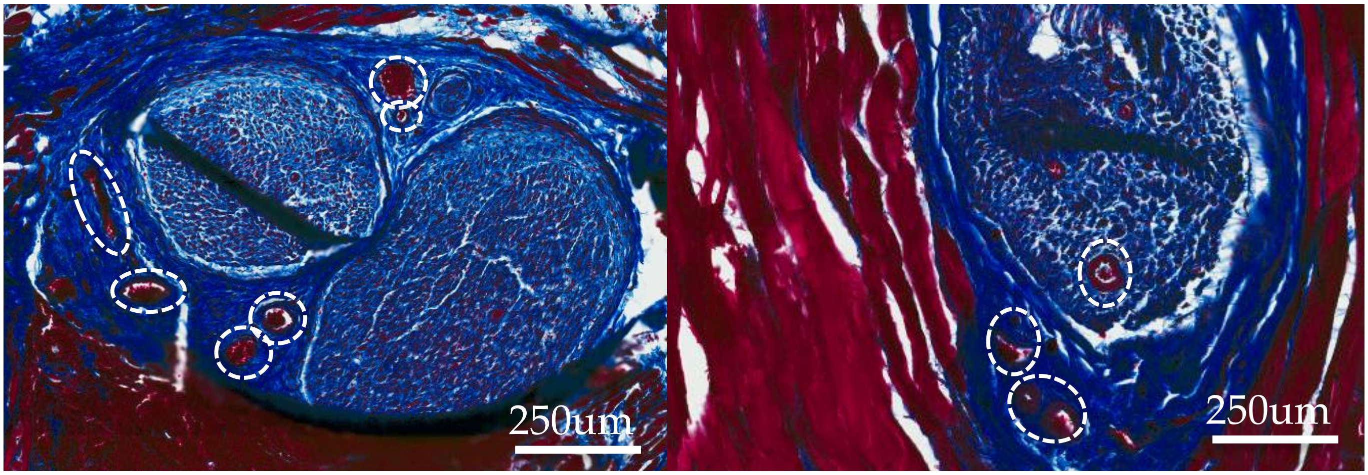

Supplemental Figure 5. Collateral angiogenesis. (Left and Right) Histological cross sections of the regenerative AMI capturing the entrance of the nerve into the graft. Numerous blood vessels (outlined in white) formed alongside the innervating nerve and were not disrupted during the second surgical stage. These vessels were critical in providing a nutrient supply during the second transfer stage and promoted the healing and overall viability of the AMI.

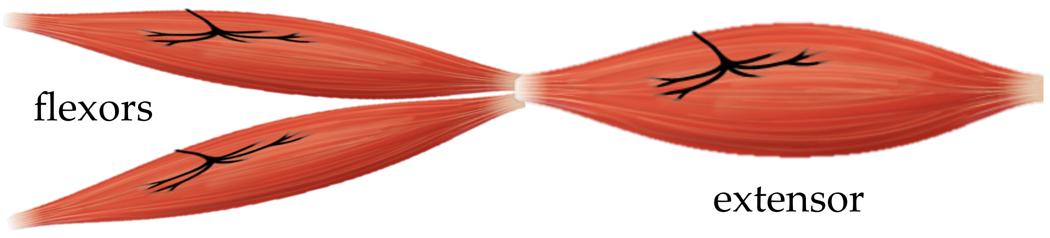

Supplemental Figure 6. Modular AMI Architecture. Given the complexity of muscle and nerve functions for certain joints, a variety of architectures can be employed to utilize muscle grafts innervated with transected nerves. Pictured is a triad architecture, which could be used for the positioning of numerous regenerative flexor-extensors grafts articulating the same joint in an AMI configuration.
